# Optimization of the chicken manure to corn straw ratio and assessment of bacterial diversity during composting

**DOI:** 10.1515/biol-2025-1306

**Published:** 2026-06-26

**Authors:** Qiuliang Yan, Xin Liu, Xiaogang Yan, Ming Li, Zhibin Ban, Xing’ai Gao, Long Chen, Zhichao Lyu, Zhonghe Li

**Affiliations:** Institute of Animal Nutrition and Feed Sciences, Jilin Academy of Agricultural Sciences, Changchun, Jilin 136100, China; Institute of Rural Energy and Ecology, Jilin Academy of Agricultural Sciences, Changchun, Jilin 136100, China; Jilin Agricultural University, Changchun, Jilin 130118, China; Jilin Province Economic Management Cadre College, Changchun, Jilin 130012, China; Institute of Grassland and Ecology Sciences, Jilin Academy of Agricultural Sciences, Changchun, Jilin 136100, China

**Keywords:** chicken manure, compost mass, microbe, predominant bacterial community

## Abstract

To enhance the quality of chicken manure and corn straw mixed compost, we evaluated the changes in physical, chemical indicators, and bacterial community diversity throughout the composting process. Three experimental groups were established with varying ratios of chicken manure to corn straw. The compost temperature was monitored daily, and samples were routinely collected to assess physical and chemical indicators. The results indicated that Group A (with a 6:4 ratio) entered the thermophilic phase earlier, sustained this phase for 20 days, exhibited faster organic matter degradation rates, and had a significantly higher total nutrient content by the end of composting compared to the other groups. The 16S rRNA high-throughput sequencing technology was utilized to investigate the alterations and distinctions in the bacterial community across 5 sampling layers of Group A samples on days 5, 15, 25 and 45. It was found that the diversity of bacterial communities initially increased, then decreased, followed by a slow increase and eventually stabilized. The predominant bacterial phyla during composting were *Firmicutes*, followed by *Actinobacteria* and *Proteobacteria. The* distribution ratio of bacteria in each sampling layer at each stage was small, while the main bacterial genera included *Bacillus*, *Corynebacterium*, *Tepidimicrobium*, *Halocella*, *Planifilum*, *Luteimonas*, and *Actinomadura*. Linear discriminant analysis revealed that the bacterial community with the greatest impact on organic matter degradation was the genus *Bacillus* (*Firmicutes*).

## 1 Introduction

The growing demand for high-quality animal products has spurred the rapid expansion of global animal husbandry. The scale of the breeding industry is expanding quickly, resulting in large quantities of livestock manure being deposited. This deposition is causing serious environmental issues, such as unpleasant odors, soil, water, and air pollution, and the spread of antibiotic-resistant pathogens, posing a significant threat to human health [1]. However, at present, much of the animal waste may not be treated safely. Livestock and poultry manures are rich in nutrients that are beneficial for plant growth. They can be aerobically degraded by microorganisms during composting under controlled conditions, which reduces their toxicity [2], 3]. Composting is a sustainable method for managing agricultural and livestock waste and protecting the environment. High-quality organic fertilizer products obtained through composting can enhance crop yields, are greener and safer than chemical fertilizers, and can optimize soil nutrient levels.

The chicken industry is one of the significant sectors within animal husbandry. Chicken manure, rich in organic matter, nitrogen, phosphorus, and potassium, serves as an excellent source of nutrients for compost fertilizer production. Research has traditionally concentrated on managing the physical and chemical parameters during aerobic composting and on the succession of microbial communities during fermentation. This approach aims to accelerate the microbial degradation of organic matter in the feces, as well as the processes of mineralization and humification, thereby reducing the toxicity of the composting product [4], 5].

Composting is a dynamic and complex biochemical process driven by the interaction of multiple microbial groups with a complex and diverse community structure. Microorganisms play a crucial role in the degradation and transformation of organic matter. Bacterial communities are widely distributed throughout various stages of composting. The bacterial community structure changes with the temperature of the stack and the degradation of the composting material. In composting systems, bacterial communities are pivotal in the breakdown of organic matter, including proteins, lipids, cellulose, and lignin, and they significantly influence the composting process and the quality of the resulting compost [6].

## 2 Materials and methods

### 2.1 Composting process and sampling

The raw materials used for composting were fresh chicken manure and corn straw. Their basic physical and chemical properties are shown in Table 1. Fresh chicken manure was obtained from the poultry breeding site, and corn straw was obtained from the corn planting base at Jilin Academy of Agricultural Sciences. After harvesting and drying, corn straw was broken into 5-cm pieces for further use.

**Table 1:** Basic physicochemical properties of compost materials.

Materials	Chicken manure (CM)	Corn straw (CS)	Group A CM: CS (6:4)	Group B CM: CS (7:3)	Group C CM: CS (8:2)
Moisture content^a^ (%)	65.04 ± 1.32	6.44 ± 0.04	–	–	–
Total carbon content^b^ (%)	35.58 ± 1.13	40.84 ± 1.59	37.68 ± 1.70	37.16 ± 1.26	36.63 ± 1.28
Total organic content^b^ (%)	61.34 ± 1.41	70.41 ± 1.69	64.97 ± 1.27	64.06 ± 1.32	63.15 ± 1.19
Total nitrogen^b^ (%)	3.12 ± 0.04	0.93 ± 0.02	2.24 ± 0.02	2.46 ± 0.04	2.68 ± 0.03
Germination index^a^ (%)	780.00 ± 2.33	115.00 ± 0.84	514.00 ± 1.84	580.50 ± 1.69	647.00 ± 1.54
C/N^b^	11.40 ± 0.21	43.92 ± 0.81	16.79 ± 0.18	15.09 ± 0.09	13.66 ± 0.08
Seed germination index^a^ (%)	34.35 ± 0.51	86.45 ± 1.41	41.34 ± 1.01	40.67 ± 0.84	38.47 ± 0.84
pH value^a^	7.37 ± 0.01	6.84 ± 0.00	7.54 ± 0.02	7.67 ± 0.01	7.81 ± 0.01

a is based on the wet weight; b is based on the dry weight.

The composting process was conducted at the Livestock and Poultry Waste Resource Utilization Center of the Jilin Academy of Agricultural Sciences, spanning 55 days from May 19 to July 13, 2023. Three composting groups were established, designated as Group A, Group B, and Group C. In each group, chicken manure and corn straw were mixed at a total weight of 5 tons, with mass ratios of 6:4, 7:3, and 8:2, respectively. Each compost pile measured 3 m in width, 1.2 m in height, and had lengths of 6 m, 4.8 m, and 3.6 m, respectively. After thorough mixing with a loader, aerobic composting fermentation was initiated. The initial moisture content of each group was adjusted to approximately 60 %. During the heating phase (days 0–10), the piles were turned once with a loader. In the thermophilic phase (days 10–20), the piles were turned three times, while during the cooling phase (days 20–30) and the maturation phase (after 30 days), the piles were turned once each.

Samples were collected on days 0, 5, 15, 25, 35, 45, and 55 during the composting process. The compost pile was divided into six equal sections along the central axis from the top, with one sampling point set at the intersection of each section, resulting in a total of five sampling points. From each point, samples were taken every 30 cm starting from the bottom, with 200 g collected each time. All samples were thoroughly mixed and then divided into two portions using the quartering method, with each portion weighing approximately 375 g. One sample was stored at 4 °C for determining moisture content, pH value, and seed germination index, while the other portion was air-dried naturally, ground into powder, sieved, and then used for measuring organic matter, total nitrogen, total phosphorus, and total potassium content.

### 2.2 Analysis of physicochemical properties of compost

#1 Temperature: The LCD-280S digital thermometer, manufactured by Tianjin Xingyuan Instrument Factory, was utilized to measure the temperature of both the reactor and the surrounding environment. Throughout the composting process, temperatures were periodically recorded at 09:00, 13:40, and 16:30. The temperature sampling points were established corresponding to the locations of the previously mentioned sample collection points. Each sampling point segmented the compost pile into three vertical sections, with the central temperature of each section being measured and the peak temperature documented. The final result was determined by taking the average of these temperature points. Moreover, the ambient temperature of the experimental facility was noted concurrently.

#2 Determination of moisture content, pH value, and seed germination index (GI)

Moisture content: The sample was dried in the 101-1 electric blast constant temperature drying box (Zhejiang Shaoxing Supper Instrument Co., Ltd.) till constant weight was obtained. The moisture content was determined by calculating the difference between the initial and final weights of the samples.

#3 pH: 5 g fresh samples were mixed with deionized water at 1:10 (W/V) ratio and added to a flask containing 45 mL sterile water. The suspension was shaken at room temperature for 30 min and allowed to stand for 30 min. The supernatant was collected for pH measurement (PHS-3E, Shanghai Magnetic Instrument Co., LTD., Shanghai, China).

#4 Seed germination index (GI): The samples were mixed with deionized water at 1:10 (W/V) ratio and were continuously shaken at 150 r/min for 1 h at room temperature. After standing for 0.5 h, the extract was collected after filter paper filtration. Subsequently, 10 mL of the extract was added to a petri dish containing 10 full cucumber seeds evenly distributed on a filter paper (according to the method mentioned in NY/T 525-2021 standard [7]). The control included the same amount of deionized water. The experiment was performed in triplicates (three petri dishes for each treatment). The dishes were incubated in the SHP-250 incubator (Shanghai Jinghong Experimental Equipment Co., LTD.) for 48 h at 25 °C. The number of germinated seeds and length of straight root were measured, and seed GI was calculated as follows.

$$\text{GI }\left(\%\right)=\left[\text{The percentage of seeds germinated in the extract culture }\left(\%\right)\times \text{The average root length of the extract culture seeds }\left(\text{mm}\right)\right]/\left[\text{The percentage of the seeds germinated in water }\left(\%\right)\times \text{The average root length of the water culture seeds }\left(\text{mm}\right)\right]\times 100\%\text{.}$$

#5 Determination of the total nutrient content and organic matter of the compost products

The total nutrient content [total nitrogen (TN), total phosphorus (TP), and total potassium (TK)] of the composting product was determined according to NY/T 525-2021, NY/T 2541-2014 [8], and NY/T 2540-2014 [9], respectively. The organic matter content was determined using the potassium dichromate volume method specified in NY/T 525-2021.

#6 DNA extraction, PCR amplification, and high-throughput sequencing

Based on the analysis of physical and chemical parameters, samples from five sampling points (#d-1-5 are upper layers, upper middle layers, middle layers, lower middle layers, and bottom layers, respectively) during the optimal group’s heating, high-temperature, cooling, and composting phases of the composting process were selected for microbial analysis. Total microbial DNA of compost samples was extracted using a soil DNA extraction kit (TIANGEN DP336, China). In total, 40 μL of DNA samples was sent to Shanghai Paronol Biotech Co., Ltd. on dry ice. The concentration and purity of the sample DNA were tested using a UV spectrophotometer and agarose gel electrophoresis, respectively. Samples with DNA amounts greater than 500 ng were considered qualified. Five eligible samples were selected for amplification of the V4 region of the 16S rRNA gene (primers: 520F: 5′-AYTGGGYDTAAAGNG-3′ and 802R: 5′-TACNVGGGTATCTAATCC-3′). The PCR-amplified products were sequenced on the Illumina Miseq sequencing platform. Two-end (paired-end) sequencing analysis was performed.

### 2.3 Sequencing data processing and bioinformatics analysis

Data were screened according to sequence quality. Low-quality sequences were removed, and operational taxonomic units (OTUs) were clustered for high-quality sequences, based on 97 % sequence similarity. Subsequently, the representative sequence of each OTU and the corresponding taxonomic information were selected using QIIME software. Based on this analysis, the abundance of OTUs, α- and β-diversities, and composition of each taxonomic level were analyzed using R software, QIIME software, and Mothur software.

### 2.4 Statistical analysis

The datasets generated and/or analyzed during this study have been released in the SRA database of the National Center for Biotechnology Information (NCBI), with the BioProject accession number PRJNA1393276. All tests were performed using three replicates, and the data obtained were statistically analyzed using SPSS 22.0 software. One-way ANOVA was performed to compare physicochemical indexes and average microbial α-diversity among treatments. For the overall temperature and humidity data, as well as the changes in total nutrients and organic matter concentration before and after composting, the Duncan multiple range test was applied to conduct significance tests at the 95 % confidence level (*p* < 0.05). For pH values and GI index data, a two-way (time and group) repeated measures ANOVA was employed. Mapping was performed using Origin 2018. RDA was performed using the Canoco 5 software to assess correlations between microbial communities and environmental factors.

**Research ethics:** Not applicable.

**Informed consent:** Not applicable.

## 3 Results and discussion

### 3.1 Temperature

**Figure 1:**
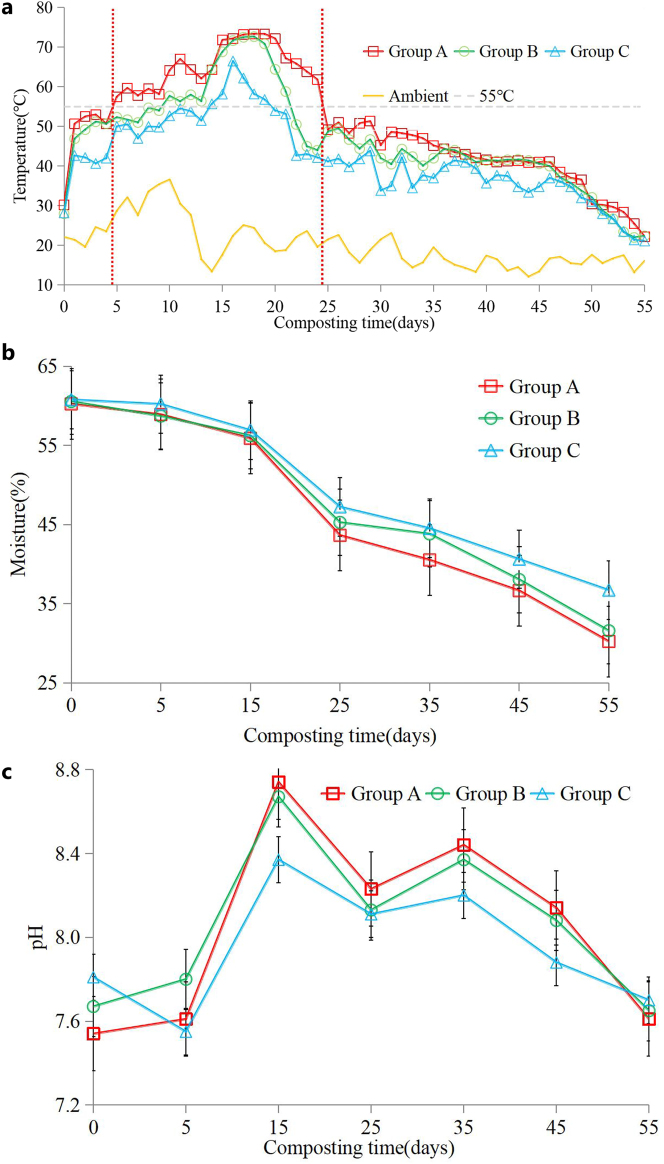
Variation in the temperature (a), moisture content (b), and pH (c) during composting.

Temperature is an important factor affecting the microbial structure during composting. The initial composting temperature rapidly increased in all groups (Figure 1(a)). After turning over the stack on day 5, the temperature in each group slightly decreased and then rapidly increased. At this time, the composting process was still in the warming period. The temperature in groups A, B, and C exceeded 60 °C on days 10, 14 and 16, respectively. The composting process entered the high-temperature period at this time. After turning over the stack on day 15, the temperature of each group rapidly increased. In groups A, B, and C, the temperatures reached the maximum on the days 17, 18 and 16 (73.3, 72.7, and 66.5 °C respectively), after which the overall composting temperature gradually decreased. From day 25, the trend of decrease in temperature slowed down, and the composting process entered the cooling period. As the composting proceeded, the temperature gradually decreased. From day 32, the composting process entered the decay period. The temperature of each group gradually decreased and reached room temperature after day 50. Overall, the composting temperature of group C was always lower than that of groups A and B. Group A exhibited the longest high-temperature period and highest temperature during composting (73.3 °C). In group A, the average cumulative temperature was 2,818.9 °C, which was significantly higher than that in groups B and C (*p* < 0.05) by 7.95 % and 20.25 %, respectively.

### 3.2 Moisture content

Moisture content is one of the important parameters of the composting process. The moisture content of all groups exhibited a gradual decreasing trend (Figure 1(b)). At the end of the experiment, the mean moisture content of Group A and Group B was 30.22 % and 31.57 % respectively, with no significant difference between the two groups (*p* > 0.05); however, the moisture content of Group C was 36.70 %, significantly higher than that of Group A and Group B (*p* < 0.05).

### 3.3 pH

Another important parameter of the composting process is pH value. Optimal pH can effectively improve microbial activity and reduce nutrient loss. Neutral or weakly alkaline pH is reported to be the most suitable for composting. The initial pH of each group was weakly alkaline (Figure 1(c)). The trend of pH change was basically the same in groups A and B. In these groups, pH steadily increased, later decreased, and became stable. In group C, the pH decreased till the 5th day but later exhibited the same trend as that observed in groups A and B. In the two-way repeated measures ANOVA (time and group), the sphericity test showed no significant deviation (*p* = 0.084). The interaction between time and group demonstrated marginal significance with a large effect size (*F*(2, 6) = 4.788, *p* = 0.057, *η* ^2^ = 0.615). Further post-hoc comparisons revealed that Group A and B exhibited slightly lower pH values than Group C at composting completion, though the difference between Groups A and B remained statistically insignificant (*p* = 0.720).

### 3.4 Total nutrients and the organic matter content

**Figure 2:**
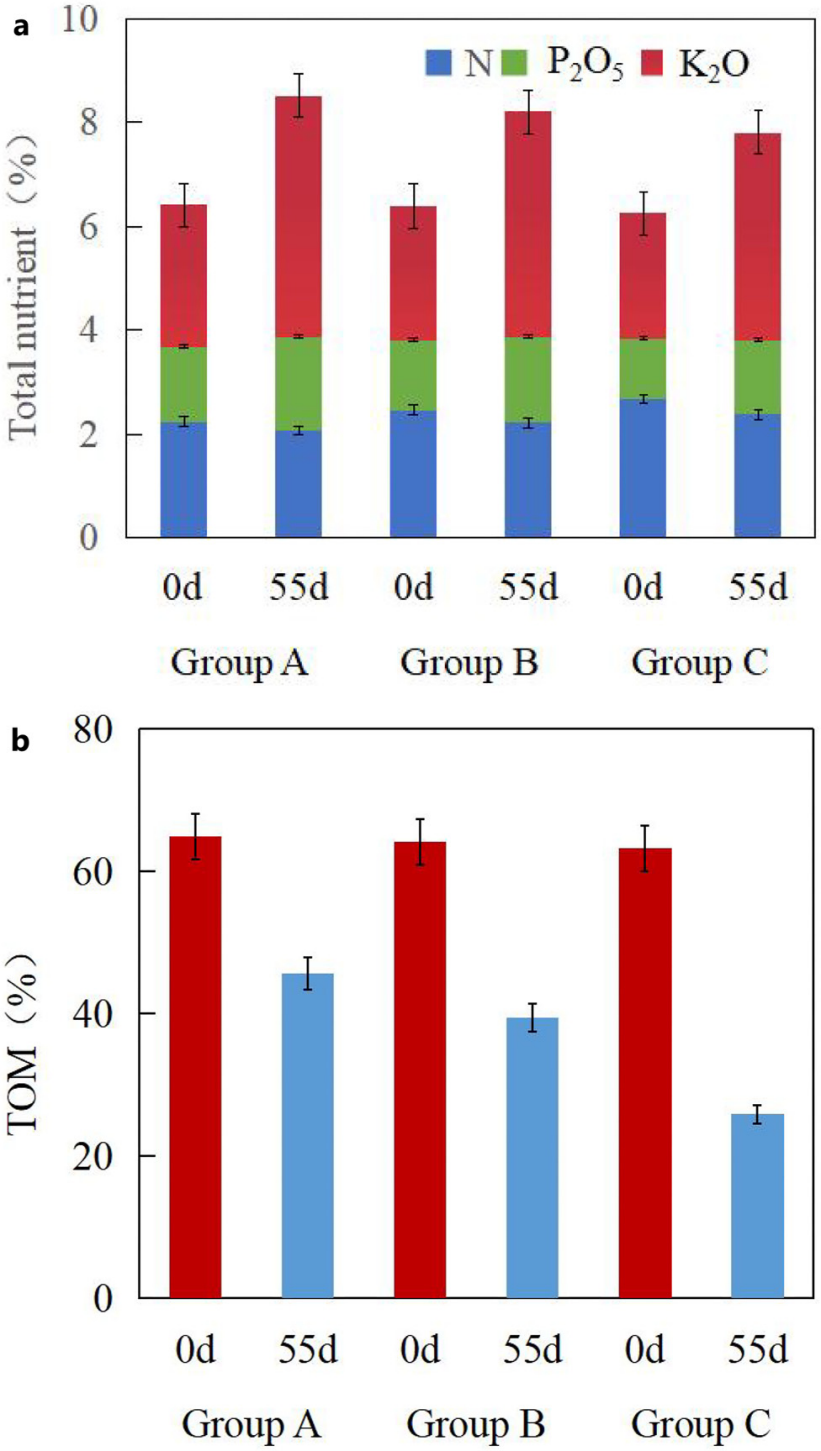
Variation in the total nutrient content (a) and total organic matter (b) during composting.

At the end of composting, the content of TN, TP, and TK in all groups increased, and the total nutrient content in them was between 7.81 % and 8.51 % (Figure 2(a) and (b)). The organic matter content of the composting products in groups A, B, and C was reduced by 29.74 %, 38.53 %, and 59.02 %, respectively, compared with the initial values. The total nutrient content and total organic matter (TOM) were significantly higher in group A than in groups B and C (*p* < 0.05).

### 3.5 GI

**Figure 3:**
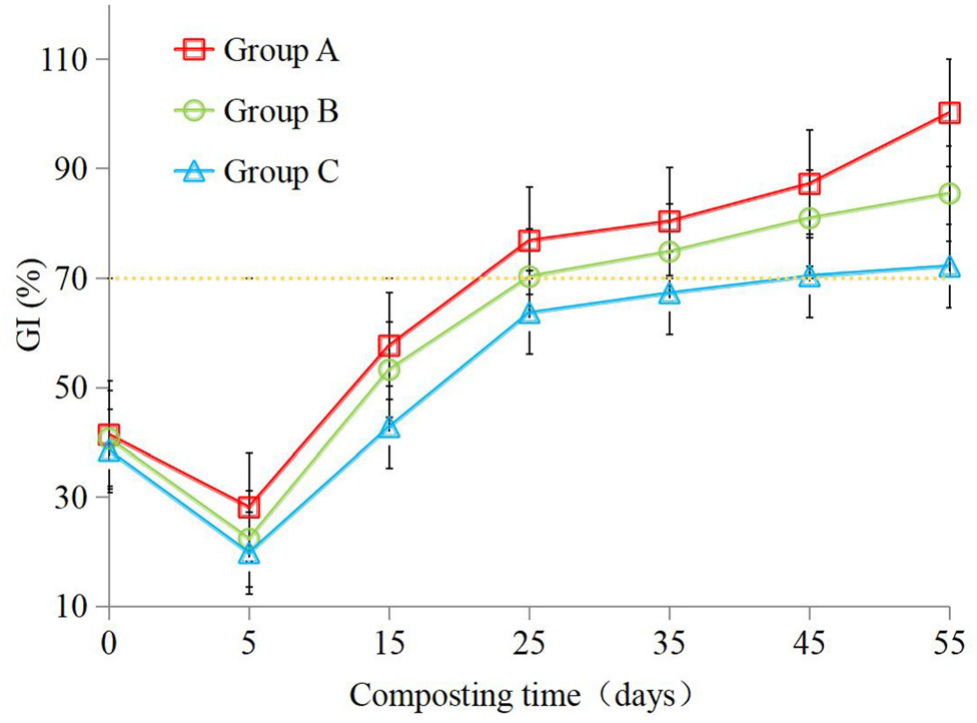
Variation in seed germination index during composting.

As the composting process progressed, the seed GI of each group showed an initial decrease followed by a gradual increase (Figure 3). The seed GI in groups A, B, and C was 76.79 %, 70.25 %, and 63.66 %, respectively, at day 25. At the end of composting, both Group A and Group B showed GI values above 85 %, while Group C had a GI of 72.14 %. A repeated measures ANOVA was conducted to examine the effects of group and time on GI. The Mauchly’s test for sphericity indicated that neither the time main effect (*W* < 0.001, *p* = 0.001) nor the time-group interaction met the sphericity assumption, so the Huynh-Feldt correction was applied. Both the time main effect and the time-group interaction were statistically significant (*p* < 0.001).

In summary, Group A initiated the thermophilic phase of composting earlier than the other groups, achieving the highest composting temperature and maintaining this elevated temperature for the longest duration. Group A exhibited a superior capability for organic matter degradation, and the total nutrient content at the conclusion of composting was notably higher in Group A compared to Groups B and C. Consequently, microbial analysis identified Group A as the representative sample group for this experiment. Based on the temperature variation curve of Group A, samples from the days 5, 15, 25, and 45 were chosen to represent the heating phase, thermophilic phase, cooling phase, and maturation phase of the composting process, respectively.

### 3.6 The succession of microbial communities during composting

#### 3.6.1 The microbial community diversities of composting products in various groups

**Figure 4:**
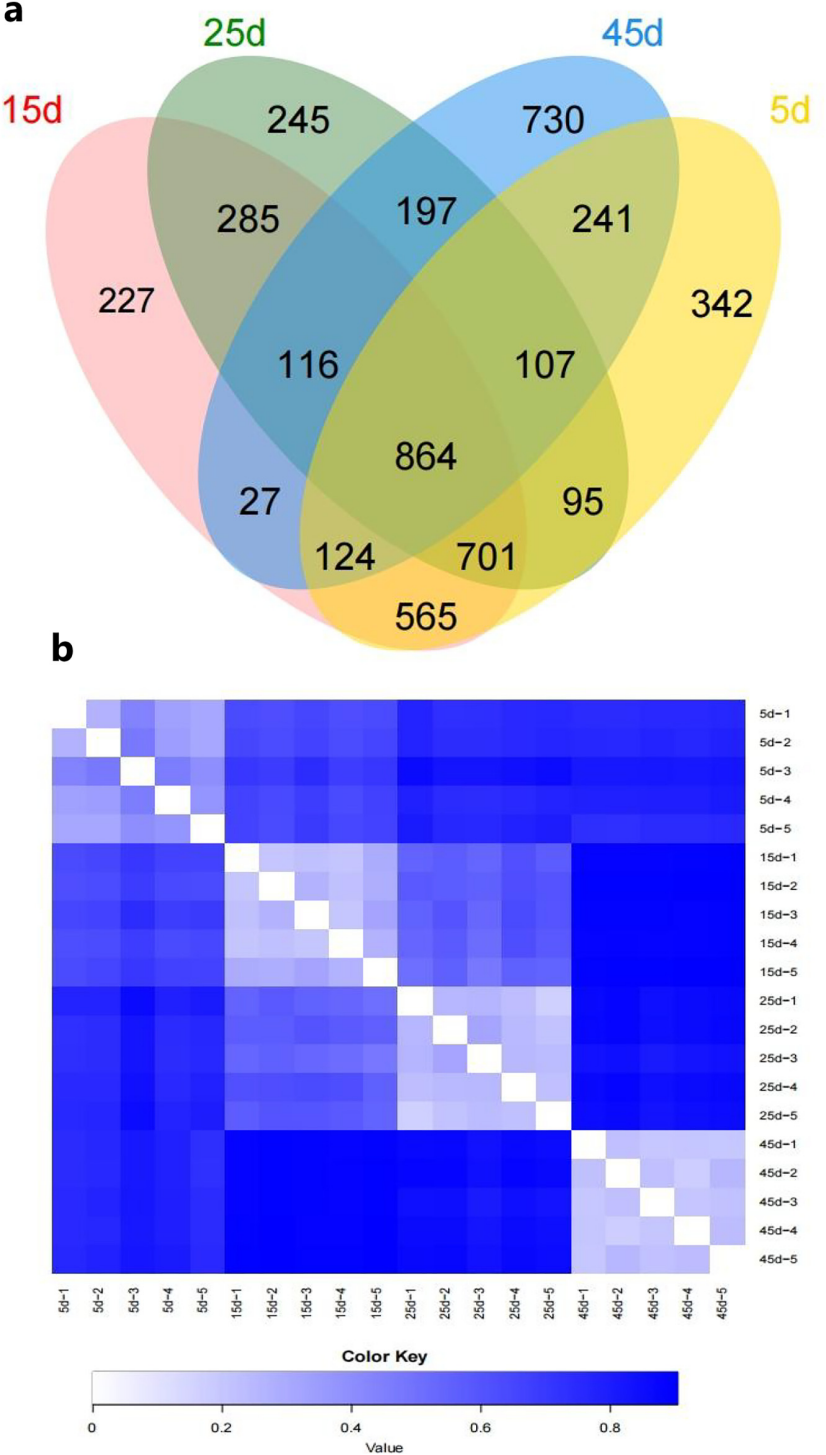

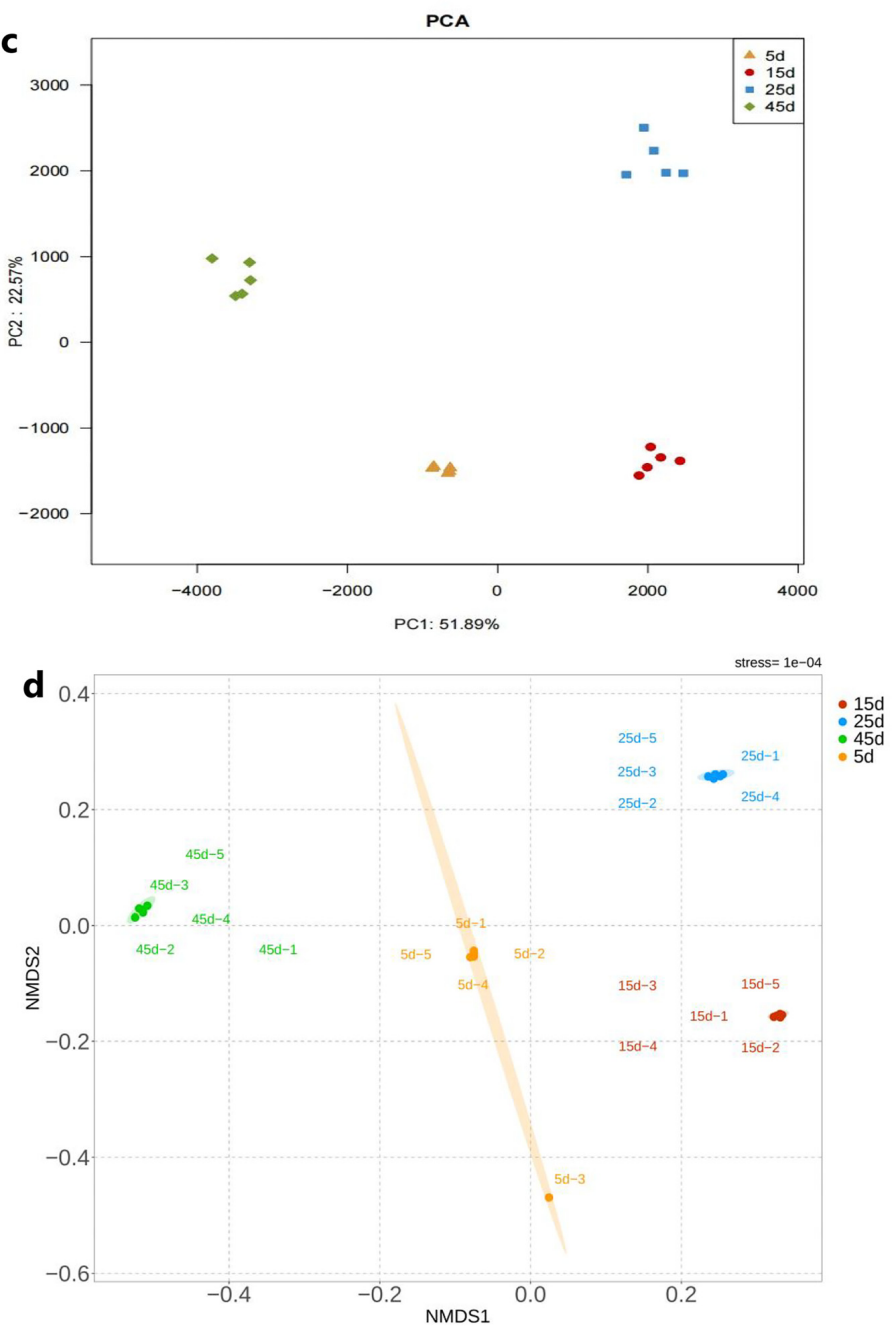
Analysis of beta diversity comparison (a) Venn diagram of OTUs, (b) heatmap analysis for distance matrix, (c) multiple samples PCA analysis, (d) NMDS analysis.

Venn plot analysis (Figure 4(a)) revealed that the number of OTUs shared across the four periods was 864, and the numbers of unique OTUs in the four periods were 342, 227, 245, and 730, respectively. The number of unique OTUs in the decay period was the highest. This number first decreased and then significantly increased during the composting period, indicating that the degradation process and changes in physical and chemical indexes significantly affected the composition of bacteria [10].

Through inter-sample comparative analysis (β-diversity analysis), we investigated bacterial diversity differences across four composting stages. The heatmap analysis in Figure 4(b) demonstrated the distance matrix between samples. Principal component analysis (PCA) (Figure 4(c)) revealed the overall distribution and key dispersion of bacterial isolates across stages. Results showed that the first principal component (PC1) accounted for 51.89 % of total bacterial variability. Bacterial community similarity was comparable between day 5 and day 15. Significant differences in bacterial diversity emerged between early and late composting stages, likely attributed to variations in environmental parameters. The Bray-Curtis distance metric quantified microbial community composition differences across composting phases, as shown in Figure 4(d), where colors represent distinct composting periods. With a significance level of 0.0001, the model demonstrated excellent fit, effectively revealing inter-sample community composition variations. The analysis revealed that, except for the 5d sample group, the distances between other period groups were relatively small, while the distances between samples from different periods were notably larger. This indicates substantial compositional shifts in bacterial communities throughout the composting process, demonstrating clear community succession.

**Figure 5:**
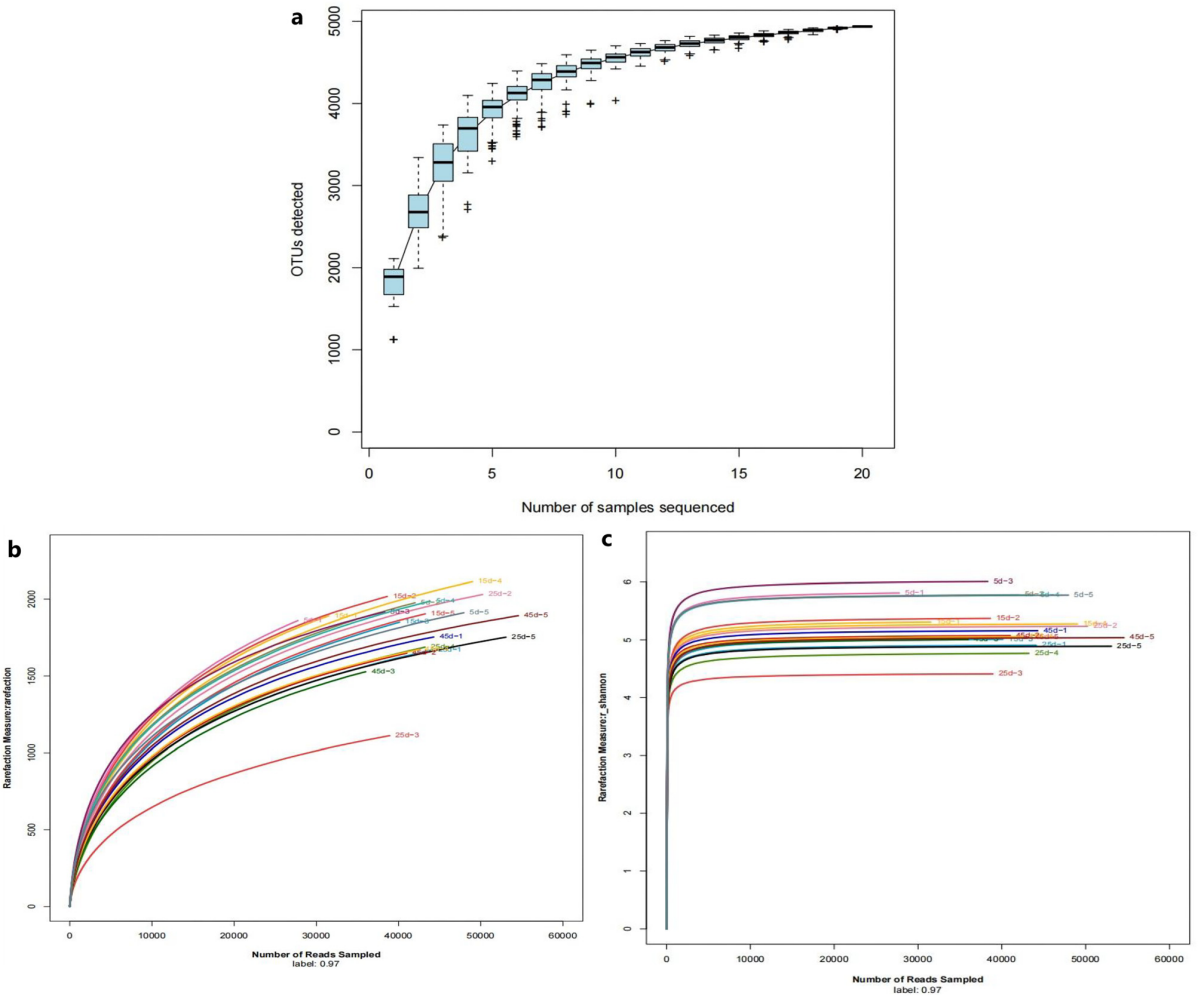
Alpha diversity analysis (a) Species accumulation curves, (b) rarefaction curve (plot of each sample at 97 % similarity levels), (c) rank-abundance distribution curve.

The number of OTUs from 20 samples gradually stabilized, indicating that the sequencing results could reasonably represent the real situation of the bacterial community in the composting process (Figure 5(a)). As more and more reads were sequenced, the rarefaction curve flattened (Figure 5(b)). This indicated that most of the bacterial gene sequences in the composting sample were detected; the sequencing results could completely represent the real bacterial community structure in the composting process, and the sequencing data could be used for further bioinformatic analysis. The level of curve at each period indicated that the proportion of each species was similar in most samples (Figure 5(c)).

Table 2 shows the bacterial α-richness and diversity, including the sequencing depth and diversity index. The species richness and diversity of bacterial communities are represented by Chao1, Simpson, and Shannon indexes. The library coverage value of each sample exceeded 98.5 %. This indicated that the sequencing depth was deep enough and the data amount was large enough to basically cover all the species information in the sample, which could represent the real distribution of bacterial communities in the composting products in this study. The Chao1 index reflects the richness of the microbial flora in the sample. The Chao1 index was higher during the warming and high-temperature period (2,472 and 2,473, respectively) than during the cooling (2,110) and decay (2,126) periods. This indicated that the richness of the microbial community was higher in the early composting period than in the late composting period. However, the Chao1 index during the warming period was not significantly different from that during the cooling and decay periods (*p* < 0.05). The microbial diversity and richness were high during the early composting period, decreased during the high-temperature and cooling periods, and increased slightly in the decay period. Therefore, according to the changes in the three indexes during the composting process, the overall diversity of the bacterial community first increased, then decreased, slowly increased, and finally tended to stabilize.

**Table 2:** Richness and diversity of bacteria in the samples at the various periods of composting.

Composting periods	Sample date (day)	Chao1 index	Simpson index	Shannon index	Coverage rate
Warming period	5	2,472 ± 84^a^	0.0086 ± 0.0010^a^	5.83 ± 0.10^a^	0.9851
High temperature period	15	2,473 ± 81^a^	0.0262 ± 0.0032^b^	5.20 ± 0.16^b^	0.9852
Cooling period	25	2,110 ± 332^b^	0.0294 ± 0.0041^b^	4.84 ± 0.30^c^	0.9893
Decay period	45	2,126 ± 98^b^	0.0252 ± 0.0042^b^	5.07 ± 0.06^bc^	0.9886

Different lowercase letters in each row indicate significant differences at *p* < 0.05.

#### 3.6.2 Phylum- and genus-level classification of bacteria in the composting products

**Figure 6:**
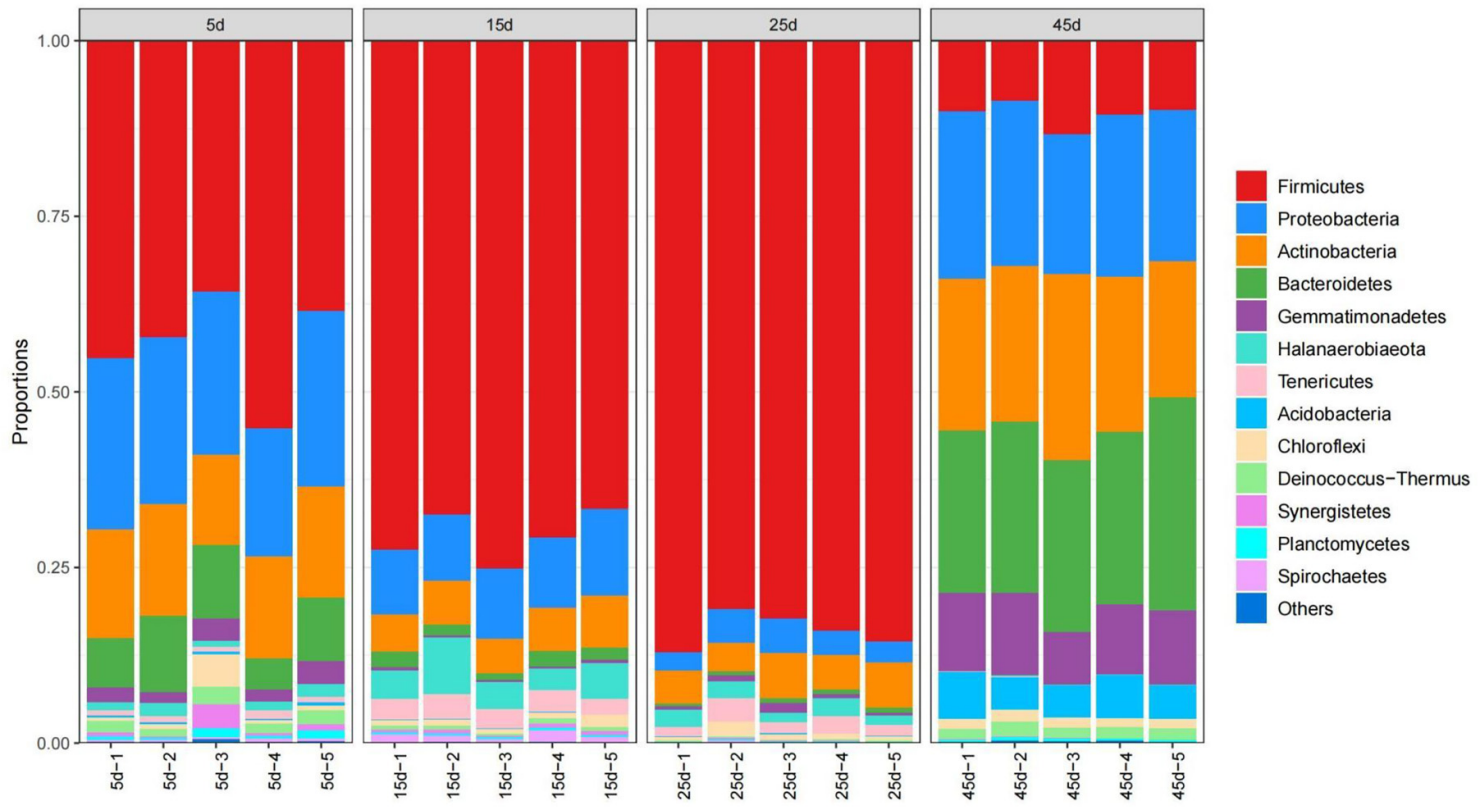
Abundance of bacterial genera in the composting samples. The genera with less than 1 % abundance are merged into “others”.

The top-13 bacterial phyla with high relative abundance in the composting samples are given in Figure 6. The phyla with low relative abundance were included in “Others.” The most abundant bacterial phyla in each group were mainly *Firmicutes*, *Proteobacteria*, *Actinobacteria* and *Bacteroidetes*. As the composting time increased, the relative abundance of *Firmicutes* decreased, and that of *Proteobacteria* and *Bacteroidetes* increased. The community structure of composting samples was similar during the high-temperature and cooling periods.

During the heating phase, the upper and middle/lower layers of the compost pile exhibited higher proportions of dominant phyla. Notably, the meso-lower layer contained 55.25 % thick-walled bacteria (Phylum Firmicutes), indicating higher concentrations of organic acids and ammonium nitrogen compared to other sections. *Proteobacteria*, a key player in organic matter degradation, accounted for 23.23 %–24.98 % across all layers during the rapid decomposition stage. *Actinobacteria* showed a higher abundance (19.39 %–26.53 %), reflecting compost maturity. *Bacteroidetes* also maintained relatively high proportions (20.14 %–30.36 %) during this phase, closely related to their specialization in breaking down refractory organic residues. Overall, the consistent phylum distribution across sampling layers demonstrated that optimal material ratios effectively regulate microbial community succession.

**Figure 7:**
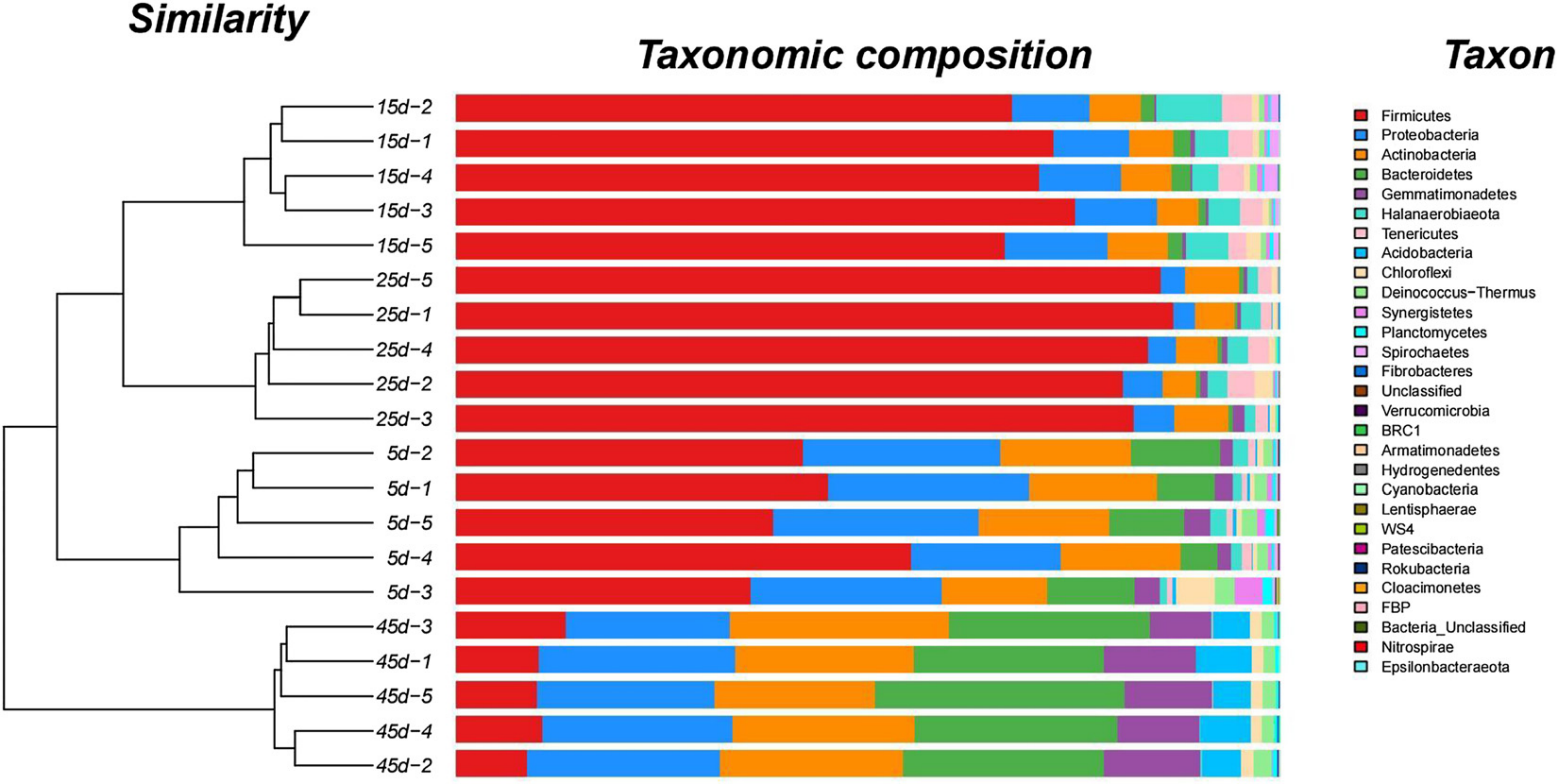
Microbial community bar plot with cluster tree.

As depicted in Figure 7, the compositions of the microbial community exhibited greater similarity at the same time point, while notable disparities were detected among the four distinct time points. The dominance of *Firmicutes* gradually diminished by day 15, whereas the proportions of *Proteobacteria*, *Bacteroidetes*, and *Actinobacteria* increased during the middle and late stages (days 25 and 45), resulting in an increase in community diversity. This suggests that the time point serves as the primary determinant of community structural alterations.

**Figure 8:**
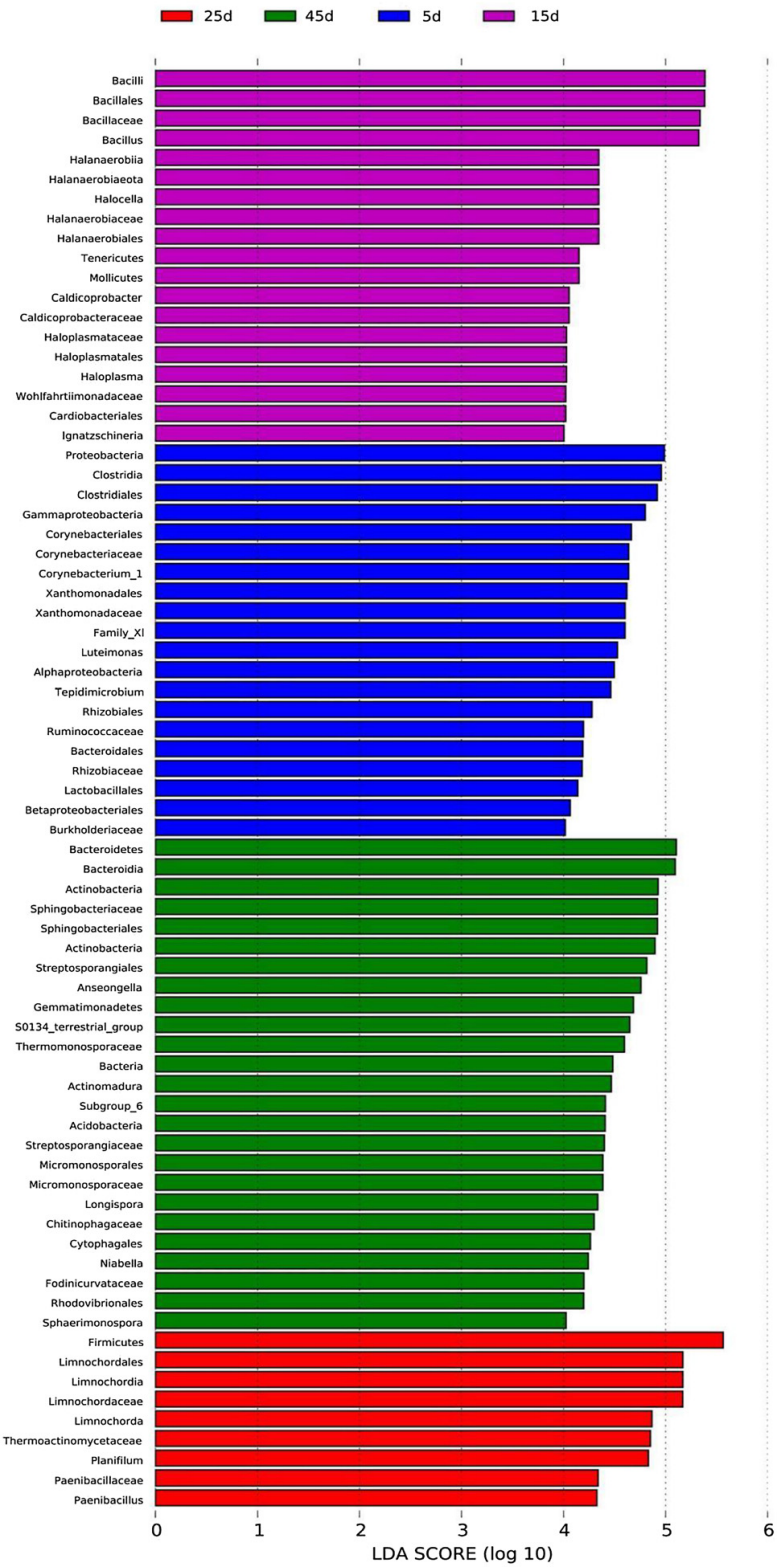
LEFSe histogram showing the LDA scores (2.0) computed to determine features at the OTU level (*p* < 0.05).

**Figure 9:**
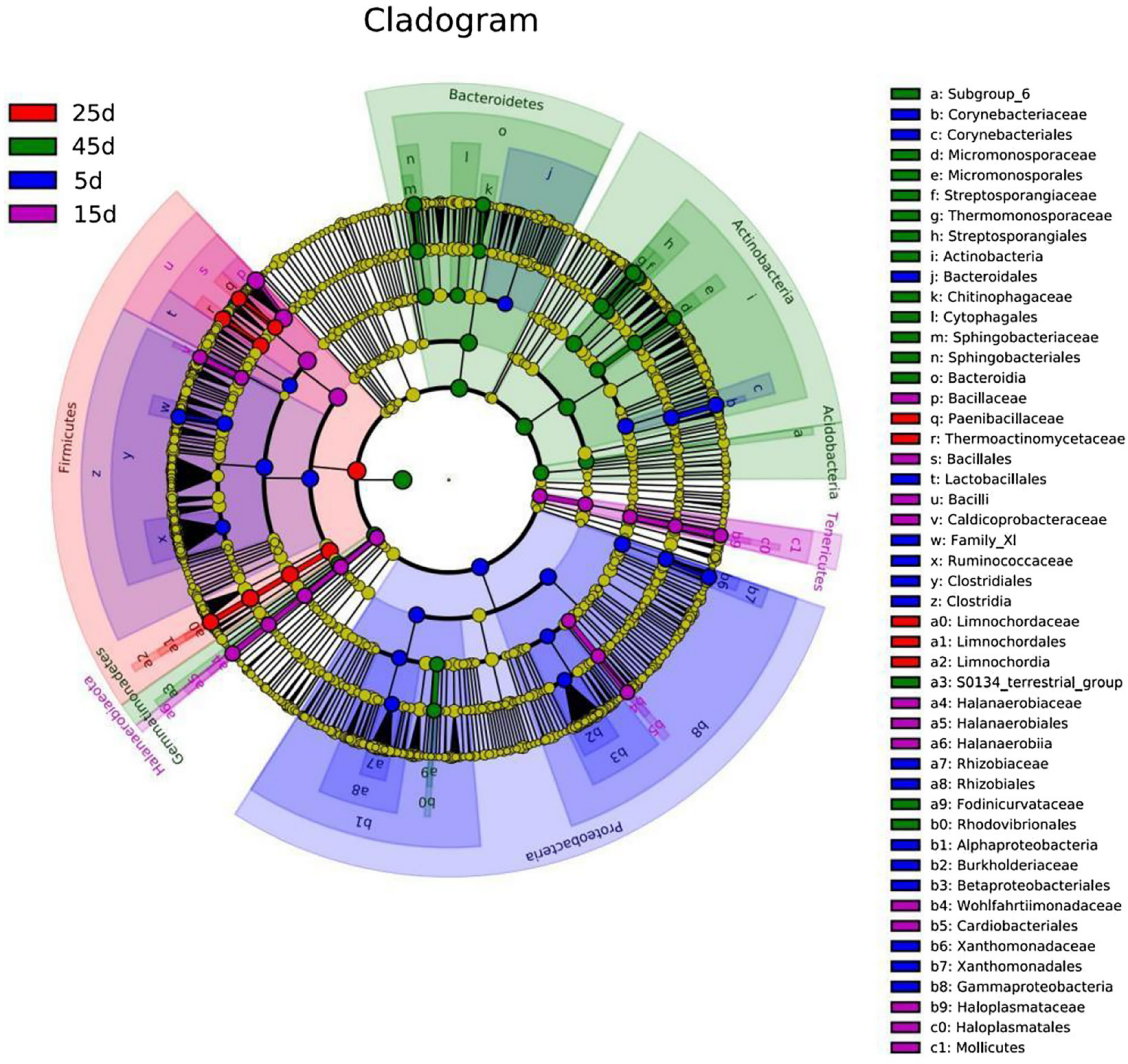
The LDA cluster tree (the species names are shown in the legend to the right).

Figure 8 shows the linear discriminant analysis (LDA) scores for the microbial taxa. The LDA threshold was set to 4 to detect species with significant differences in abundance between various composting periods. In total, 20, 19, 9, and 25 indicator bacteria (characteristic bacterial groups) were detected in the warming, high-temperature, cooling, and decay periods, respectively. Combined with Figure 9, it is found that in the warming period, *Rhizobium* (*Rhizobiales*, *Rhizobiaceae*), *β-Proteobacteria* (*Betaproteobacteriales*, *Burkholderiaceae*), and *Clostriobacteria* (*Clostridiales*) were the indicator bacteria. During the high-temperature period, *Bacillaceae* (*Paenibacillaceae*), *Thermoactinomycetaceae*, *Bacillus* (*Bacillales*), *Haloplasmataceae* (*Haloplasmatales*), and *Halanaerobiaceae* (*Halanaerobiales*, anaerobic halophile) were the characteristic bacterial communities. The cooling period exhibited the lowest number of indicator bacteria, with only *Firmicutes* and *Limnochordaceae* (*Limnochordales*).

The decay period exhibited the highest number of indicator bacteria: *Sphingomonas* (*Sphingomonadaceae*), *Sphingolipidomonas* (*Sphingomonadales*), *Cytophagales* and *Streptosporangiaceae* (*Streptosporangiales*), and *Micromonosporaceae* (*Micromonosporales*).

In conclusion, samples gathered at four different time points demonstrated distinct clustering patterns on the phylogenetic tree, and notable alterations were detected in the dominant taxonomic units. The trend of changes in the dominant phyla is in line with the aforementioned conclusions.

## 4 Discussion

Temperature can directly reflect the degradation of organic matter by microorganisms in the process of composting, and is also an important indicator of the success of composting [11]. Each group in this study underwent four periods according to the change in temperature: warming, high-temperature, cooling, and decay periods. The temperature in groups A, B, and C exceeded 55 °C after the 5th, 10th, and 14th day, respectively. Groups A, B, and C were in the high-temperature period for 20, 12, and 6 days, respectively. Comparatively, group C entered the high-temperature period later, and the duration of this period was shorter. Interestingly, the ambient temperature decreased after the 10th day. However, the composting temperature of all groups was still on an upward trend. All groups exhibited a rapid increase in temperature; however, compared with group A, the duration of maintenance of high temperature was shorter in the other groups. This indicated that the composting temperature was less affected by the external environmental temperature in group A than in groups B and C. This may be because the raw material ratio in group A was more suitable for maintaining high temperature and species diversity, and the rate of microbial degradation of organic matter was higher, because of which the released heat maintained the high temperature for a long time. In the early composting period, the temperature in each group exhibited an increase for a short period, followed by a slow decrease. Groups A and B exhibited a more obvious upward trend in terms of temperature in this period than group C. This indicates that the stacking operation can increase the oxygen content of the stack to avoid excessive temperature, provide ambient temperature for the growth of microorganisms, and promote aerobic degradation of organic matter. Moreover, C/N was high in groups A and B; thus, sufficient carbon source was available for the composting process. After each stacking operation, the microbial life activity was enhanced. And the degradation rate was accelerated, and the exothermic period was extended. Van-Truc Nguyen et al. [12] reported that when the C/N ratio was approximately 20, the appropriate stack turning frequency was more helpful to provide more stable and favorable conditions for the growth and the degradation activity of microorganisms in the compost. When the chicken manure to corn straw volume ratio was 6:4, the composting product reached the highest temperature quicker and maintained that temperature for longer. In the decay period, the temperature change after stack turning was relatively small, indicating poor availability of organic matter for microbial decomposition, which cannot release heat in a short time.

The water in the stack is an essential carrier for the decomposition of organic matter by microorganisms. Poor moisture content will lead to the incomplete conversion of organic matter in the stack and increase the toxicity of the composting product. Very high moisture content will not only promote the anaerobic environment in the stack but also lead to leachate overflow and loss of nutrients, resulting in secondary pollution. Li et al. [13] reported that low humidity promotes the reduction of CH_4_ and H_2_S, while high humidity decreases NH_3_ and N_2_O levels. Moderate humidity, however, optimizes both maturation degree and humification efficiency. Li et al. [14] used chicken manure, rice bran, and rice straw as raw materials for composting and reported that at the initial moisture content of 50 %–55 %, the composting temperature and nutrient value of the composting products were higher. Therefore, determination of the initial moisture content is important, and it may be related to the composition of the raw material for composting. In this study, the moisture content of the three groups decreased as the temperature increased in the early period of composting. It was below 45 % when the temperature approached room temperature, meeting the requirements of NY/T 3442-2019 [15] compost product quality standards.

The pH value reflects the acidity or basicity of the environment in the composting process. The initial pH of each group was different because corn straw is weakly acidic; therefore, the pH of the group containing more corn straw was relatively low. In the early periods of composting, the increase in pH was less obvious or the pH even slightly decreased. This is because the high temperature during the early period of composting promoted microbial ammonification reaction, releasing significant amounts of NH_3_, and organic matter degradation promoted the release of organic and inorganic acids and CO_2_ [16]. And the acids released by nitrification will lead to a drop in pH. In the subsequent periods, as a large amount of organic matter is decomposed, the ammonification reaction is promoted, and the pH value will rise significantly. However, in the decay period, the pH of the three groups exhibited a trend of slow decrease. This was mainly due to the enhanced activity of nitrifier bacteria, enhanced nitrification reaction, and weakened ammonification. At the end of the composting process, the pH of each group was 7.61–7.70, meeting the requirement of weak alkaline pH for composting products.

The total nutrient content of compost products is a key indicator of the quality of compost. High nutrient content can not only promote the healthy growth of crops but also improve their ability to adapt to external environmental conditions. Due to the gradual decomposition of the organic matter in the stack during the composting process, the gases volatilize. It results in the decrease of the total mass of the stack, which will also lead to the false increase in the total nutrient content. Awasthi et al. [17] reported that the TP and TK contents gradually increased as the composting progressed, which may be related to the enrichment effect of organic matter degradation. At the end of the composting process, the final content and rate of increase of TN, TP, and TK were significantly higher in group A than in groups B and C (*p* < 0.05). The rate of increase of total nutrient content in group A (45.97 %) was higher than that in groups B and C by 4.59 % and 6.75 %, respectively. The nutrient content in group C was the lowest, indicating that the ratio of raw materials ultimately affects the nutrient content of compost products. However, the final nutrient content of each group exceeded the requirements of NY/T 525-2021 (4.0 %), indicating that the compost products can be used as organic fertilizer to improve soil fertility and quality. The ratio of raw materials in group A was more conducive to producing good-quality compost.

TOM is the sum of carbon-containing organic compounds present in various forms. In the warming and high-temperature periods of composting, the easily degradable organic matter in the stack is preferentially used by microorganisms, and redegradable organic matter is used in the late compost period, which eventually leads to the decline in TOM [18], 19]. The TOM content was significantly lower in group C than in other groups (*p* < 0.05); moreover, the rate of decrease of TOM was the highest in group C. This indicated that an appropriate ratio of raw materials could provide a suitable C/N ratio to accelerate the effective degradation of TOM and prevent the carbon loss caused by the release of greenhouse gases such as CO_2_ and CH_4_. This has a positive impact on the quality of compost [20]. Tang et al. [21] demonstrated that the C/N ratio has the most significant impact on nitrogen loss, whereas water content and oxygen concentration have minimal effects on carbon and nitrogen loss; however, there is a close correlation between these two factors. Overall, 85 % of the initial TN can be used during microbial degradation, and 70 % of the effective carbon is lost as carbon dioxide during the carbon curing process. The final TOM in groups A and B was above 30 %, meeting the standard requirements of NY/T 525-2021.

The GI can reflect the toxicity of compost products. A large number of small molecular organic acids and base cations are generated due to the incomplete degradation by some microorganisms [22], which may cause improper seed germination. Therefore, the GI value was lower during the early composting period. Further, group A exhibited a more prominent trend of increase in GI values than groups B and C. This is because the addition of corn straw reduces the concentration of base cations and increases the porosity among the stacks. As the composting proceeds, microbial activity is facilitated under aerobic conditions; the organic matter degradation efficiency is increased, and generation of toxic substances is reduced [23]. The continued high temperature can eliminate harmful substances such as weed seeds and pest eggs. Therefore, as composting proceeds in a well-controlled environment, the content of toxic substances in the compost gradually decreases, and the GI value gradually increases [24]. At the end of the composting, the GI value of each group exceeded 70 %, reaching the standard requirements of NY/T 525-2021. This indicated that the composting products were no longer toxic for plant growth.

In this study, we used high-throughput sequencing technology to reveal the changes in bacterial communities in chicken manure and corn straw during aerobic composting. The diversity indexes and abundance of bacteria tended to first increase and then decrease throughout the composting process. During the warming period, sufficient organic material and a suitable growth environment are conducive to the growth and metabolism of bacteria, resulting in a significant increase in bacterial diversity and abundance. During the high-temperature period, some thermophilic and heat-resistant microorganisms were dominant, leading to decreased bacterial diversity. This trend was consistent with the results of Liu et al. and Zhang et al. [25], 26]. After the high-temperature period, the degradable organic matter content in the raw material sharply decreases, resulting in a significant decrease in the bacterial diversity and abundance in the cooling period. In the decay period, as the temperature drops, microbial activity slows down, and the bacterial community structure gradually stabilizes. It can be inferred that the succession of microbial communities and changes in metabolic function during composting are largely controlled by the environmental changes caused by dominant communities. Bao et al. [27] reported that organic matter content and composting temperature had a significant effect on the change of the microbial communities during various composting periods. Therefore, the composition and degradation function of microbial communities in livestock manures should be further studied to promote good-quality compost production.

In this study, *Firmicutes*, *Proteobacteria*, *Actinobacteria*, and *Bacteroidetes* were the dominant bacterial phyla in the compost product. They represented over 80 % of the total identified sequences. This is consistent with previous studies suggesting that these phyla play a major role in the degradation of organic matter in composting [28]. The *Firmicutes* and *Proteobacteria* were the two phyla with the largest relative abundance. *Firmicutes* can effectively use carbohydrates, sugars, and some easily degradable organic matter to obtain sufficient nutrition through the decomposition and fermentation process. In addition, *Firmicutes* can form endospores; therefore, they are tolerant to adverse conditions and can survive in unsuitable environments. This is the main reason why *Firmicutes* become the dominant bacteria in the composting warming period and high temperature periods [29]. *Proteobacteria* is the largest group of bacteria, with various functions such as lignin degradation and nitrogen fixation, and it was distributed during all periods of composting [30]. *Actinomyces* and *Bacteroidetes* dominated during the decay period. This is because the easily degradable organic material has been exhausted at this time, while the refractory cellulose and lignin content is high. *Actinobacteria* can effectively degrade cellulose, hemicellulose, and lignin. Many bacteria from *Bacteroidetes* can degrade large molecular organic matter. In this study, the addition of corn straw led to the increase in cellulose content, providing sufficient nutrients for the growth and metabolism of *Bacteroidetes*. Therefore, *Actinobacteria* and *Bacteroidetes* dominated in the late compost period, which is consistent with the study by Zhang et al. [31].

An analysis of the microbial community distribution across five sampling layers indicated minimal variation in the four dominant bacterial groups at each stage. Research by Yin et al. [32] demonstrated that during composting processes involving pig manure and straw, aerobic bacterial genera progressively transitioned to anaerobic ones from the top to the bottom of the compost pile. Although the addition of straw increases aerobic microbial dominance, this study observed minimal variation in bacterial distribution across compost layers. The relatively uniform microbial composition after pile turning and densification likely resulted from standardized operational procedures. These findings confirm that straw incorporation and rigorous composting management effectively enhance the safety and decomposition efficiency of manure composting.

The dominant genera in the composting process have been shown to be widespread in nature and man-made environments, including soil and sewage treatment systems, as well as multiple composting systems. *Bacillus* has obvious advantages in the composting process as it can produce spores to resist the external high-temperature environment. Consequently, the elimination of mesophilic bacteria during the high-temperature period leads to an increase in the relative abundance of *Bacillus* [33], 34]. The results of the dominant genera observed in the apotrophic decay period were generally consistent with those of the dominant phyla and indicated that *Actinobacteria* played an important role in the apotrophic decay process.

*Clostridium* is the biomarker of the initial period of composting. It is widely distributed in nature, especially in the soil. Most *Clostridium* species can hydrolyze sugars and proteins. In addition, *Clostridium* can produce spores, enhancing its resistance to the external environment. The abundance of *Clostridiales* was significantly higher in the initial composting period than in other periods. Therefore, *Clostridiales* was an indicator of the initial period of composting. *Bacillus* order in biomarker in the high temperature period is involved in most of the transformation reaction to organic matter at high temperature, and can also accelerate the degradation of cellulose and lignocellulose. Moreover, their resistance to high temperature increases their abundance in the high-temperature period. During the decay period, *Actinomycetes* can decay plant polymers at the appropriate temperature. Therefore, *Actinosaprophytesmycetales* played an important role in promoting the compost decay process and were the indicator bacteria at this period [35]. These observations were consistent with the results of the dominant phyla, class, and genera in the composting periods.

The results of microbial community succession indicate that aerobic microorganisms predominate at each treatment stage. Although most studies suggest that the addition of straw does not inherently affect greenhouse gas emissions, scientific formulations and meticulous management can effectively suppress localized anaerobic environments. This method reduces methane and nitrous oxide production from anaerobic conditions, thereby indirectly mitigating the impact of manure management on global warming potential.

## 5 Conclusions

Chicken manure mixed with corn straw at a 6:4 ratio (m/m) can increase composting temperature and prolong the duration of high temperatures; this indicates that a higher proportion of straw accelerates the degradation of TOM in the compost pile, reduces nutrient loss and harmful gas emissions, decreases the toxicity of the compost product, and improves compost quality.

*Firmicutes*, *Proteobacteria*, *Actinobacteria,* and *Bacteroidetes* were the dominant phyla throughout the composting process. The distribution of bacterial genera at each compost level was limited, and aerobic microorganisms were the dominant microbial group. Certain *Bacillus* species demonstrated advantages during the high-temperature phase, while *Actinobacteria* thrived during the cooling and decay phases, serving as indicator microorganisms for these periods. *Clostridiales*, *Bacillus,* and *Actinomycetales* are indicative of the initial, high-temperature, and decay phases of composting, respectively. It has been shown that regulating straw and managing the composting process can ensure an aerobic environment for the compost and enhance the decomposition of the final product.

## Supplementary Material

Supplementary Material
