# Supplementary material for: Optimization of the chicken manure to corn straw ratio and assessment of bacterial diversity during composting
Source: Open Life Sci. 2026 Jun 26;21(1):20251306. doi: 10.1515/biol-2025-1306 (PMC13307393; doi:10.1515/biol-2025-1306)
Supplement: Supplementary file 1 — Supplementary Material [file j_biol-2025-1306_suppl_001.pdf]

supplementary information

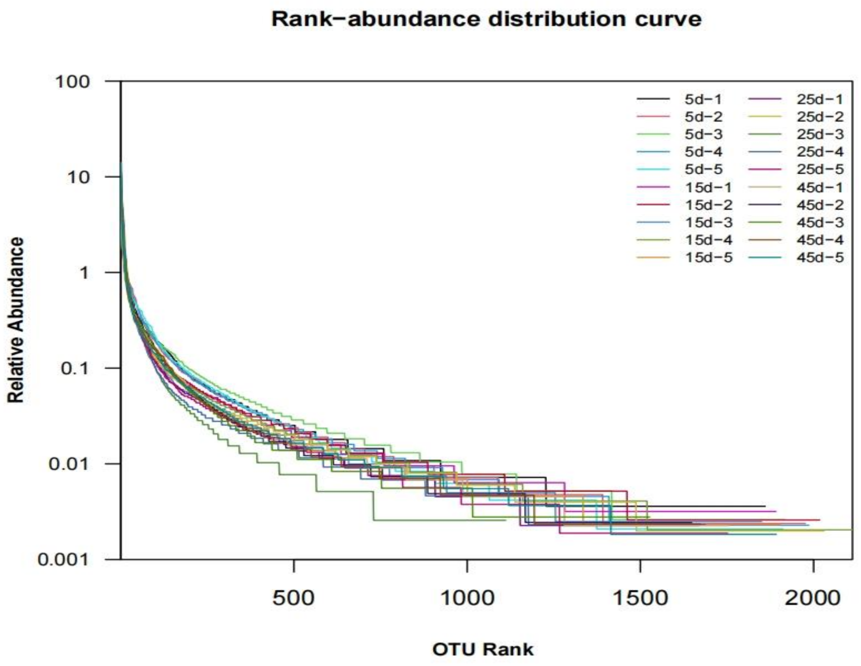

Figure 1 . Shannon Wiener curves of samples

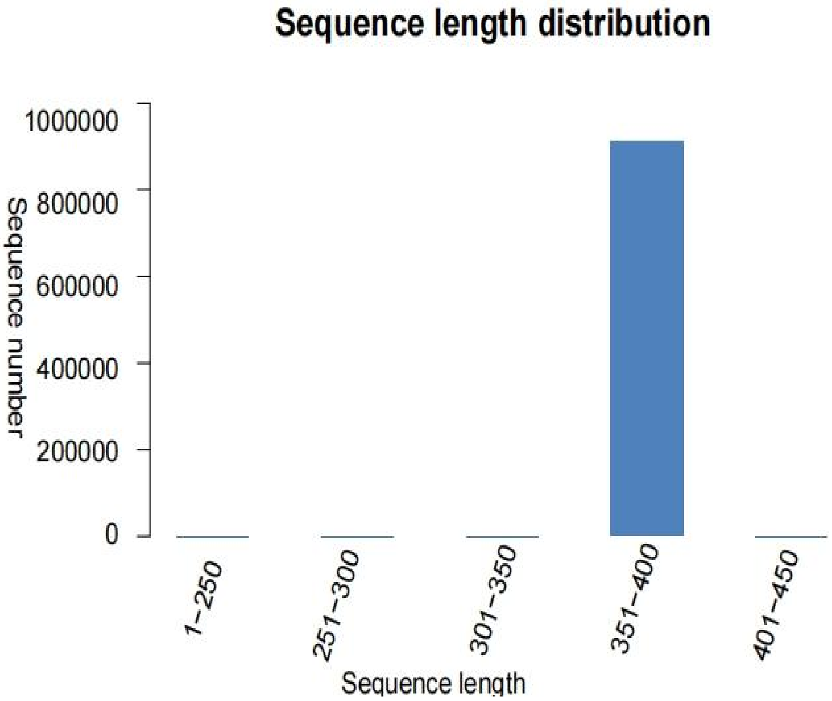

Figure 2 . Distribution of the length of trimmed sequences (almost all the sequences were approximately of length 351–400 bp);
